# Editorial: Plant epigenetics and chromatin dynamics - EPIPLANT 2021-2022

**DOI:** 10.3389/fpls.2023.1260391

**Published:** 2023-11-08

**Authors:** Guillaume Moissiard, Marie Mirouze, Cristel C. Carles, Clémentine Vitte

**Affiliations:** ^1^ Laboratoire Génome et Développement des Plantes - Unité Mixte de Recherche (LGDP-UMR)5096, Centre National de la Recherche Scientifique (CNRS), Perpignan, France; ^2^ Laboratoire Génome et Développement des Plantes - Unité Mixte de Recherche (LGDP-UMR)5096, Université de Perpignan Via Domitia, Perpignan, France; ^3^ Institut de Recherche pour le Développement (IRD), Équipe Mixte de Recherche (EMR)269 Mechanisms of Adaptation and Genomics (MANGO) Centre National de la Recherche Scientifique (CNRS)/Institut de Recherche Pour le Développement (IRD)/Université de Perpignan Via Domitia (UPVD), Perpignan, France; ^4^ Grenoble Alpes University – Centre National de la Recherche Scientifique (CNRS) – Institut national de recherche pour l'agriculture, l'alimentation et l'environnement (INRAE) – Commissariat à L'énergie Atomique et aux Énergies Alternatives (CEA), Plant and Cell Physiology Lab (LPCV), lnstitut de Recherche Interdisciplinaire de Grenoble - Département de Biologie Structurale et Cellulaire Intégrée (IRIG-DBSCI), Commissariat à l’Énergie Atomique et aux Énergies Alternatives (CEA), Grenoble, France; ^5^ Université Paris-Saclay, Institut National de Recherche pour l’Agriculture, l’Alimentation et l’Environnement (INRAE), Centre National de la Recherche Scientifique (CNRS), AgroParisTech, Génétique quantitative et évolution (GQE) – Le Moulon, Gif-sur-Yvette, France

**Keywords:** EPIPLANT, chromatin, epigenetics, histone modifications, DNA methylation

For decades, plants have been considered as organisms of choice to study chromatin dynamics and epigenetic modifications, such as DNA methylation and post-translational modifications of histone proteins. Plants can display very complex genomes, varying tremendously in size from one species to another, and displaying very sophisticated epigenetic pathways involved in various chromatin-related processes regulating gene expression, repressing DNA repeats such as transposable elements (TEs) or controlling genome stability. All these processes play crucial roles to ensure proper plant development and appropriate responses to an ever-changing environment.

The Research Topic ‘*Plant Epigenetics and Chromatin Dynamics - EPIPLANT 2021-2022’* was set in the frame of the two later EPIPLANT meetings held in 2021 and 2022, which gathered scientists studying molecular and evolutionary aspects of plant epigenetics and chromatin dynamics. This Research Topic compiles one review article, one mini-review article and five original research articles describing various aspects of chromatin-related processes.

In their review, Jeynes-Cupper and Catoni give an exhaustive summary of long-distance signaling of RNA molecules, such as small RNA (sRNAs) that are involved in post-transcriptional gene silencing (PTGS) by cleavage or translational inhibition of RNA targets, as well as in transcriptional gene silencing (TGS) through the deposition of epigenetic modifications in a sequence-specific manner. This aspect of long-distance RNA movement is put into the context of plant grafting, given the importance of this horticultural technique for crop production and improvement.

Velay et al. review the phenomenon of chromatin barrier that restrains heterochromatin spreading to insulate euchromatin regions from transcriptional repression. They discuss the importance of active DNA demethylation at TE boundaries to maintain the proper expression of adjacent genes. They also review the function of Arabidopsis topoisomerase VI (Topo VI) in maintaining the proper expression of genes located in euchromatin islands that are surrounded by repressive heterochromatic blocks. They finally consider the potential connections between the chromatin barrier, DNA looping and insulator elements involved in the three-dimensional organization of genomes [like the formation of topological-associated domains (TAD)] or regulation of enhancer-promoter interaction in plants.

In their original research article, Meschichi et al. describe the ANCHOR system, also known as ParB/ParS, which is an elegant fluorescent protein (FP)-based DNA-labeling tool allowing real-time monitoring of a single locus subnuclear localization *in planta*. The ANCHOR system, which is derived from bacteria, can be used to determine the subnuclear localization and dynamics of a single locus in various plant tissues, with different ploidy levels, or at particular stages of the cell cycle. Using a combination of FPs, the ANCHOR system can ultimately allow the detection of several loci in the same nucleus, which will be of major interest for further understanding of chromatin dynamics at single locus resolution.

In *Chlamydomonas reinhardtii*, the MUT9p kinase phosphorylates histone H3 at threonine 3 to fulfill transgene and TE silencing. The Arabidopsis genome encodes four MUT9p-like kinase homologs that have been named MLKs. In their research article, Huang et al. investigate the genetic interaction between the Arabidopsis *mlk* mutants. They show that MLKs act redundantly to regulate flowering time through the phosphorylation of H2A at serine 95 (H2AS95) and, as previously shown for MLK4, MLK3 interacts with Circadian Clock Associated 1 (CCA1), which is a master regulator of the circadian clock in plants.

Using immuno-labelling with fluorescent *in situ* hybridization (immuno-FISH) coupled to ultrastructural super-resolution microscopy, Karimi-Ashtiyani et al. describe in their research article the epigenetic determinism of the hybrid wheat-rye centromere of a reconstructed chromosome 1B. They found that the CENH3 histone variant, a marker of centromere activity, is only partially incorporated at the 1BL/1RS centric translocation. Furthermore, the authors noticed that only the centromeric regions deriving from rye did incorporate CENH3 of wheat into the 1BL/1RS hybrid.

RNA-directed DNA methylation (RdDM) is an important epigenetic pathway involving specialized enzymes that collectively repress TEs. DEFECTIVE IN RNA-DIRECTED DNA METHYLATION (DRD1), DEFECTIVE IN MERISTEM SILENCING 3 (DMS3) and RNA-DIRECTED DNA METHYLATION 1 (RDM1) form the DDR complex, which plays a pivotal role in the RdDM pathway. In their research article, Niu et al. investigate the mechanisms by which the DDR complex regulates the transcription and transposition of the *ATCOPIA78/ONSEN* TE family, which is reactivated upon heat treatment. The authors also confirm that *ONSEN* transcription and transposition can be uncoupled in several epigenetic mutant combinations, with the *drd1* mutant showing the strongest transposition phenotype in comparison to *dms3* and *rdm1* mutants.

In Arabidopsis, CURLY LEAF (CLF) is a histone methyl-transferase involved in the deposition of H3K27me3 to repress developmentally-regulated genes. In the last research article of this Research Topic, Nugroho et al. characterize the function of the CLF homolog in *Brassica rapa*, which is called BrCLF. The authors show that as in Arabidopsis, BrCLF is important for the regulation of developmental processes such as plant organ development and floral transition. They also uncover that BrCLF plays a role in stress-related metabolic pathways.

In conclusion, this Research Topic covers a broad range of chromatin-related processes including chromatin dynamics, small RNA-mediated regulation of gene expression, subnuclear organization of chromatin, TE biology and post-translational modification of histones. Considering the importance of epigenetic pathways involved in plant response to challenging environments, it illustrates the pertinence of studying these molecular processes in the context of climate breakdown.

## Author contributions

GM: Writing – original draft. MM: Writing – review & editing. CC: Writing – review & editing. CV: Writing – review & editing.

